# Hydroxy‐α‐Sanshools From *Zanthoxylum bungeanum* Maxim. Alleviate Obesity in Mice via the Regulation of Appetite and Gut Microbiota

**DOI:** 10.1002/fsn3.71952

**Published:** 2026-06-01

**Authors:** Tianting Luo, Yuping Zhu, Fangyan Xu, Jiao Xie, Huifang Chen, Likang Qin, Tingyuan Ren

**Affiliations:** ^1^ College of Brewing and Food Engineering Guizhou University Guiyang P. R. China; ^2^ School of Basic Medicine Guizhou Medical University Guiyang P. R. China; ^3^ Tongren Preschool Education College Tongren P. R. China; ^4^ Key Laboratory of Environmental Pollution Monitoring and Disease Control Guizhou Medical University Guiyang P. R. China; ^5^ College of Public Health Guizhou University of Traditional Chinese Medicine Guiyang P. R. China

**Keywords:** dietary bioactive compounds, gut–brain axis, high‐fat diet, hydroxy‐α‐sanshool, *Zanthoxylum bungeanum* Maxim

## Abstract

To explore whether hydroxy‐alpha‐sanshool (HAS) prevents obesity by modulating appetite and the gut microbiome, an obesity model was established using mice fed a high‐fat diet. The food intake, body mass, feed efficiency and organ index of the mice were recorded, and the serum levels of GLP‐1 and PYY were measured. The mRNA and protein expression of appetite‐related genes in the small intestine and brain tissue were detected by real‐time quantitative PCR and Western blotting, and the intestinal flora was analyzed by 16S rRNA gene sequencing. The results revealed that the body weight, food intake and feed efficiency of the mice were significantly reduced (4.28%, 14.46%, and 25.33%, respectively) and that the levels of GLP‐1 and PYY were significantly increased (*p* < 0.05) (9.11% and 5.49%, respectively) by the HAS intervention. The relative mRNA expression of NPY and AGRP in the small intestine decreased significantly (39.62%, 21.95%) (*p* < 0.05); the relative mRNA levels of CART, GLP‐1R, NPY2R and GLP‐1 in the brain tissue increased significantly; and those of NPY and AGRP decreased significantly (*p* < 0.05). The protein expression of POMC and GLP‐1 increased significantly (*p* < 0.05), whereas the protein expression of NPY and AGRP decreased significantly (*p* < 0.05). The diversity of intestinal flora in the cecal contents and the relative abundance of probiotics among the dominant flora increased. In conclusion, HAS could modulate the expression of appetite‐related factors and the composition of intestinal microbiota in mice fed with a high‐fat diet by regulating food intake and maintaining energy homeostasis, which is consistent with the involvement of the gut‐brain axis in this regulatory mechanism.

## Graphical abstract

Hydroxy‐alpha‐sanshool could modulate the expression of appetite‐related factors and the composition of intestinal microbiota in mice fed with a high‐fat diet by regulating food intake and maintaining energy homeostasis, which is consistent with the involvement of the gut‐brain axis in this regulatory mechanism.

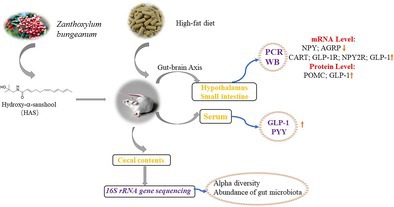

**Abbreviations**
AGRP: agouti‐related protein
CART: cocaine and amphetamine regulated transcript
GLP‐1: glucagon‐like‐peptide 1
GLP‐1R: glucagon‐like peptide‐1 receptor
NPY: neuropeptide Y
NPY2R: neuropeptide Y receptor Y2
POMC: pro‐opiomelanocortin
PYY: peptide YY

## 1 Introduction

Obesity is a global epidemic, the incidence of which has increased because of improvements in quality of life, a severe lack of exercise, and the intake of high‐fat, high‐calorie foods (Li, Chen, et al. 2024). Since 1990, the global rate of human obesity has more than doubled, and the rate of adolescent obesity has tripled. As of 2022, more than 390 million children and adolescents aged 5 to 19 years are classified as overweight, including 160 million who are considered obese (WHO 2025). Furthermore, obesity contributes to the development and progression of numerous comorbidities, including non‐alcoholic fatty liver disease (Polyzos et al. 2019), obstructive sleep apnea (Gnessi et al. 2012), hormone imbalance‐related malignancies (Friedenreich et al. 2020), various cardiovascular diseases (Duflou et al. 1995), and osteoarthritis (Kulkarni et al. 2016). In obese individuals, disrupted metabolic homeostasis and impaired function of multiple organ systems (e.g., the immune system) elicit chronic low‐grade inflammation, which in turn accelerates the pathogenesis of a wide spectrum of secondary disorders (Castro et al. 2017).

Consequently, the global prevalence of obesity has become an important issue. At present, the principal methodologies employed in the effective treatment of obesity are drug therapy and surgery. Nevertheless, these approaches carry risks of rebound weight gain and severe side effects (Ren et al. 2023). Consequently, there is an urgent need to explore natural products containing dietary bioactive compounds as adjunctive therapies for obesity. Furthermore, previous studies have focused primarily on the mechanisms through which HAS improves obesity through the AMPK‐HIF1‐PKM2 pathway and through the regulation of AMPK/SIRT‐1/PPARγ signaling (Ren et al. 2023; Zhang et al. 2023). However, at present, no available studies have investigated whether HAS exerts anti‐obesity effects through the gut‐brain axis.

The “gut–brain axis” (GBA) constitutes a complex bidirectional communication network, the basic structure of which consists primarily of the autonomic nervous system (Liu et al. 2022), the central nervous system, the neuroendocrine system, and the immune system (Jiang et al. 2024). In mammals, the gastrointestinal tract and the brain have been shown to regulate food intake and certain physiological processes, including secretion, digestion, and absorption. This regulation is achieved through the transfer of information via a feedback loop, thus maintaining energy homeostasis (Blanco et al. 2021). Energy homeostasis is maintained through the harmonization of three factors: energy intake (EI), energy expenditure (EE), and energy storage (ES). Disruption of this delicate balance, resulting in a state of energy imbalance, is a hallmark of obesity (Piaggi et al. 2022). The hypothalamus, a pivotal brain center that regulates energy homeostasis, is closely related to the onset and progression of obesity (Seong et al. 2019). The gut microbiota is composed of trillions of microorganisms that inhabit the gastrointestinal tract (Gomes et al. 2018). Numerous studies have demonstrated a relationship between the composition of the gut microbiota and the development of obesity and metabolic syndrome in humans (Li et al. 2025; Virtue et al. 2019). Genetically obese mice possess a gut microbiota that fosters increased energy intake, and research has indicated that a diet high in fat can lead to reversible alterations in the distal gut microbiome (Wang et al. 2019). Consequently, the gut microbiota may represent a novel therapeutic target for the treatment and prevention of obesity and metabolic syndrome.

*Zanthoxylum bungeanum* Maxim. is a fruit belonging to the peppercorn genus and is categorized within the *Rutaceae family*. It has a long history in China, where it has been utilized as both a culinary flavoring and a component of medicinal formulations. Hydroxy‐α‐sanshool (HAS) is a naturally occurring active component in peppercorns. HAS is found in the skin of the fruit, as well as in the fruit itself and the leaves of the plant (Li et al. 2020). HAS is among the major components of *Zanthoxylum alkylamides* (ZAs) and is a significant contributor to the flavor of *Zanthoxylum* (Li, Ren, et al. 2024). On the basis of our previous research, ZA has been shown to decrease the synthesis of blood lipids, liver fat, and cholesterol in rats fed a high‐fat diet. In addition, it has been shown to enhance the dysfunctional metabolism of sugar, fat, and protein in individuals with type I diabetes (Ren, Zhu, Xia, et al. 2017; Ren, Zhu, and Kan 2017; You et al. 2015). Moreover, ZA enhances hepatic lipid and energy metabolism dysfunction via the AMPK‐HIF1‐PKM2 pathway. In addition, it has been shown to activate the Nrf2/HO‐1 signaling pathway and reduce oxidative damage induced by a high‐fat diet (Ren et al. 2023). However, research on the role of hydroxy‐α‐sanshool in relation to gut health is relatively limited, and the precise mechanisms through which it exerts a regulatory effect on appetite via the metabolic pathways of gut microorganisms remain to be elucidated.

Based on our previous research findings, the present study aims to investigate the effects of hydroxy‐α‐sanshool on high‐fat diet‐induced obesity in terms of growth status, appetite, and gut microbiota. This work is expected to provide novel insights for the application and promotion of hydroxy‐α‐sanshool, as well as for the adjuvant treatment of obesity using natural dietary bioactive components.

## 2 Materials and Methods

### 2.1 Chemicals

HAS was purchased from Shanghai Yuanye Biotechnology Co. Ltd. (Shanghai, China; CAS No. 83883‐10‐7 and article number B26430; HPLC ≥ 98%; Purity detection of HAS, Figure S1). Soya bean oil (food grade) was procured from Shanghai Jinlongyu Food Co. Ltd. (Shanghai, China); egg yolk powder (food grade) was purchased from Henan Wanbang Chemical Technology Co. Ltd. (Henan, China); and both cholesterol and cholate (food grade) were purchased from Guangzhou Jiaxing Food Ingredients Co. The mouse GLP‐1 ELISA (F10563) kit and mouse PYY ELISA kit were procured from Shanghai Qiaodu Biotechnology Co. Phosphorylated protease inhibitor, a BCA protein quantification kit, and an SDS–PAGE gel preparation kit were obtained from Wuhan Xavier Biotechnology Co. (Hubei, China). All other chemicals were reagent grade.

### 2.2 Preparation of Hydroxy‐α‐Sanshool (HAS) Gavage Doses

As evidenced by earlier research, the administration of HAS at doses ranging from 2 to 12 mg/kg‐bw has been shown to impact lipid, glucose, and protein metabolism in murine models, with a heightened influence on lipid and protein metabolism observed at a dose of 8 mg/kg‐bw in particular (Wang et al. 2019; Wei et al. 2021; You et al. 2015). Consequently, the dosage of HAS selected for this study was 8 mg/kg·bw. The body weight of the mice was gavaged at a volume of 1 mL/100 g. The HAS standard was accurately weighed and dissolved in edible soybean oil (Lu et al. 2021), and the HAS solution was prepared at a concentration of 0.8 mg/mL.

### 2.3 Animals

Thirty Kunming male mice (4 w, 20–22 g) were maintained in a well‐ventilated environment. The room temperature was maintained at 23°C ± 2°C, the relative humidity was set at 45%–65%, and a 12‐h day–night alternation was implemented. All animal experiments were approved by the Experimental Animal Ethics Committee of Guizhou University and conducted in accordance with Chinese animal welfare guidelines. The experimental protocol was approved by the Experimental Animal Ethics Committee of Guizhou University (ethical approval number EAE‐GZU‐2020‐P003).

After one week of basal chow, the mice were randomly divided into a control group (CG) (*n* = 10) and a high‐fat chow group (*n* = 20) on the basis of body mass. The CG group was fed a basal diet, and the high‐fat group was fed a high‐fat diet (78.8% basal, 10% lard, 10% egg yolk powder, 1% cholesterol, and 0.2% bile acids). In accordance with the literature (Chen 2020), the high‐fat feed was formulated in the laboratory. After eight weeks of feeding, the mice in the high‐fat group were randomly divided into a high‐fat model group (MG) and an HAS dose group (8 mg/kg‐bw (DG)) according to their body mass. Each group contained 10 mice. In accordance with the stipulated gavage dose of 1 mL/100 g of mouse body weight, the blank group and the high‐fat model group were administered equivalent quantities of edible soybean oil by gavage on a daily basis between 9:00 and 11:00, while the dose group was given HAS solution by gavage (Lu et al. 2021). During the experimental phase, the mice were provided ad libitum access to food and water, and the quantity of food and body mass of each group were meticulously documented on a weekly basis. The gavage volume was adjusted on a body mass basis, and the gavage was conducted continuously over a period of four weeks.

### 2.4 Sampling and Analytical Procedures

At the conclusion of the experimental cycle, following a 12‐h fast, blood was extracted from the orbits of the mice by ether anesthesia. This sample was then transferred into a centrifuge tube and subjected to centrifugation at 4°C and 4000 r/min for 15 min. Thereafter, the samples were transferred to a storage container maintained at −80°C. The mice were euthanized by cervical dislocation, after which the liver, kidneys, heart, spleen, brain, small intestine, cecum, and other tissues were expeditiously dissected on an ice pack. The blood on the surface of the tissues was meticulously removed using physiological saline, and the area was then wiped clean with absorbent paper. The weights of the organs were recorded. The middle approximately 1 cm of small intestinal tissue and one‐quarter of the cerebral tissue were meticulously selected and placed in paraformaldehyde fixative for histopathological examination. The remaining small intestine and brain tissues were divided into three portions and placed in RNA‐free tubes. These tissues were then transferred to a −80°C refrigerator after quick freezing with liquid nitrogen for subsequent determination of relevant gene expression. The cecal tissues were then meticulously hollowed out under aseptic conditions, and the contents of the cecum were placed in centrifuge tubes. These samples were then quickly frozen in liquid nitrogen and subsequently transferred to a refrigerator set at −80°C for further intestinal 16S rRNA gene sequencing assays.

### 2.5 Determination of Feed Efficiency

On the basis of the body mass and food intake of the mice during the experiment, the feed efficiency of the mice was calculated with the following equation (Ren and Kan 2018):

(1) $$\text{Feed efficiency}\;\left(g/100\;g\right)=\frac{\text{weight increase of mice}\;\left(g\right)}{\text{food intake}\;\left(g\right)}\times 100$$

### 2.6 Determination of Organ Indices

On the basis of the body mass and organ weight of the mice, the organ and tissue indices were calculated with the following equation (Lu et al. 2021):

(2) $$\text{Organ index}\;\left(\%\right)=\frac{\text{Organ weight}\;\left(g\right)}{\text{body mass}\;\left(g\right)}\times 100$$

### 2.7 Determination of Serum Intestinal Hormone Levels in Mice

The serum levels of glucagon‐like peptide 1 (GLP‐1) and peptide YY (PYY) were determined by means of enzyme‐linked immunoassay (ELISA), with the specific procedures being executed in strict adherence to the guidelines stipulated within the kit instructions. GLP‐1 and Peptide YY were obtained from Shanghai Qiaodu Biotechnology Co.

### 2.8 RNA Extraction and Quantitative Real‐Time PCR (RT–qPCR) Analysis

Total RNA was extracted from the brain tissue and reverse transcribed to obtain cDNA, and the concentration and purity of the RNA were measured with a NanoDrop 2000. The relative mRNA expression levels of the genes FFAR2, AGRP, NPY, CART, POMC, NPY2R, GLP‐1R, GLP‐1, and PYY were detected by fluorescence quantitative polymerase chain reaction, with GAPDH as the internal reference gene. The relative expression levels of each gene in the different groups were calculated using the 2^−ΔΔCT^ method to determine the relative expression levels of the mRNA of each gene (Lu et al. 2021). Table 1 shows the primer sequences and product lengths for each gene.

**TABLE 1:** Primer sequences and product lengths of related genes.

Gene name	Primer sequence (5′‐3′)		Product size
	Forward primer	Reverse primer	
GAPDH	CCTCGTCCCGTAGACAAAATG	TGAGGTCAATGAAGGGGTCGT	133
FFAR2	GGCACTGAGAACCAAATAACCTG	GAAGCGCCAATAACAGAAGATG	138
AGRP	TGTGTAAGGCTGCACGAGTCC	GGCATTGAAGAAGCGGCAGT	87
NPY	TCCGCTCTGCGACACTACATC	AAGGGTCTTCAAGCCTTGTTCT	136
CART	AGAAGGAGCTGATCGAAGCGT	AGAATTGCAGGAAGTTCCTCG	182
POMC	TTCCTGGCAACGGAGATGAA	ACTCGGCTCTGGACTGCCAT	170
NPY2R	TTACCATCAGCGAAGGCACAA	CTGTCGATGTCCACAGCGAGT	111
GLP‐1R	GCTATCGGCGTCAACTTTCTT	TCGTCCATCACAAAGGCAAA	173
GLP‐1	GGGACCTTTACCAGTGATGTGAG	CAGAATGGTGCTCATCTCGTCA	177
PYY	GTCGCAATGCTGCTAATCCTG	GGACATCTCTTTTTCCATACCGC	175

### 2.9 Western Blotting (WB) Analyses

Approximately 100 mg of brain tissue was extracted from the experimental and control groups. The tissue was then frozen in liquid nitrogen, after which 1 mL of precooled SDS lysate and 10 μL of PMSF were added. The mixture was thoroughly agitated, refrigerated for 40 min, and then removed and shaken 2–3 times, after which the tubes were placed on a centrifuge for 20 min at 4°C (Chen et al. 2022). The top layer, or the supernatant, was then mixed with 5× protein sampling buffer at a ratio of 1:4 by volume. The tubes were then placed back on the centrifuge and boiled for 5–10 min. The protein concentration of the resulting fluid was measured using a commercially available BCA protein assay kit (Servicebio, Hubei, China). To ensure experimental standardization, β‐actin was selected as the reference protein in the present study (Zhang et al. 2022). This protein is stably expressed in the brain tissues of mice under different dietary intervention conditions, making it suitable for standardization calibration in Western blot assays. Equal amounts of protein samples (30 μg/well) were separated by 12% sodium dodecyl sulfate–polyacrylamide gel electrophoresis (SDS–PAGE) and transferred to a polyvinylidene difluoride (PVDF) membrane for further analysis. The PVDF membrane was then immersed for a period of 2 h at room temperature in a solution of 5% skim milk powder dissolved in a Tween 20 (TBST) solution. This was followed by low‐speed shaking for an additional hour and then an overnight incubation at 4°C. Thereafter, the membrane was subjected to an additional 2 h of incubation at 4°C. On the following day, the membrane was washed three times with TBST solution for five minutes each. Afterward, the membrane was incubated with the appropriate secondary antibody for a period of two hours at room temperature. The membrane was then subjected to another three washes with TBST. In the darkroom environment, ECL Developer A and Developer B solutions were prepared at a volumetric ratio of 1:1, ensuring immediate operational readiness. The PVDF membrane was subsequently meticulously transferred onto filter paper to expel residual water droplets. Afterward, it was carefully positioned within the designated development compartment of the gel imaging system. The developer was added to the PVDF membrane in equal amounts using a pipette gun, and the membrane was then covered with plastic film. Following a waiting period to allow the developer to react, the membrane was exposed to develop the image. Quantity One software was used to analyze the grayscale values of the target bands, and protein expression was calculated according to the method described by Ren, Zhu, and Kan (2017).

### 2.10 16S rRNA Gene Amplicon Sequencing

Total genomic DNA was extracted from the cecum contents of mice in the blank group, high‐fat model group, and HAS dose group using an OMEGA Soil DNA kit. The quantity and quality of the extracted DNA were measured by means of a NanoDrop NC2000 spectrophotometer and agarose gel electrophoresis, respectively. The V3–V4 region of the 16S rRNA gene was subjected to PCR amplification using the forward primer 338F (5′‐ACTCCTACGGGGAGGCAGCA‐3′) and reverse primer 806R (5′‐GGACTACHVGGGTWTCTAAT‐3′). The sample‐specific 7‐bp barcodes were combined into primers for multiplex sequencing. 16S rRNA sequencing libraries were constructed and analyzed after the acquisition of informative sequences through Illumina MiSeq sequencing and the execution of quality control measures, deconjugation, low‐quality filtration, and the elimination of chimeric sequences from the original materials. In this experiment, 16S rRNA gene sequencing was carried out with the assistance of Sichuan Panomics, and the sequence data were analyzed primarily with QIIME 2 software (2019.4) and R software (4.0.2). Detailed experimental procedures and data processing methods are presented in Supporting Information S2.

### 2.11 Pathological Examination

The hematoxylin–eosin (HE) method was employed to stabilize mouse brain and small intestine tissues. After fixation, pruning, dehydration, and embedding were completed. After the tissues were sliced, the following operations were carried out: dewaxing the slices in water; hematoxylin staining; flushing with tap water; differentiation with hydrochloric acid alcohol; flushing with tap water; placement of the slices in warm water at 50°C or a weakly alkaline aqueous solution to restore the blue color until the blue color appeared; flushing with tap water; adding 85% alcohol; eosin staining; washing with water for 3–5 s; dehydration with a gradient of alcohol; xylene transparency; and sealing of the slices with neutral gum. The specimens were processed according to the SOP for pathological examination. Finally, microscopic observation and photographic analysis were carried out.

### 2.12 Statistical Analysis

The statistical analysis was conducted using SPSS version 23.0 and Origin version 2019b. The experimental data are presented as the mean ± standard deviation (SD). Group differences were analyzed by one‐way ANOVA, followed by LSD and Duncan's post hoc tests for multiple pairwise comparisons to control the Type I error rate. Two‐tailed *t*‐tests were used where appropriate. A value of *p* < 0.05 was considered statistically significant.

## 3 Results and Analysis

### 3.1 Effect of HAS on the Growth of High‐Fat Diet‐Fed Mice

**FIGURE 1:**
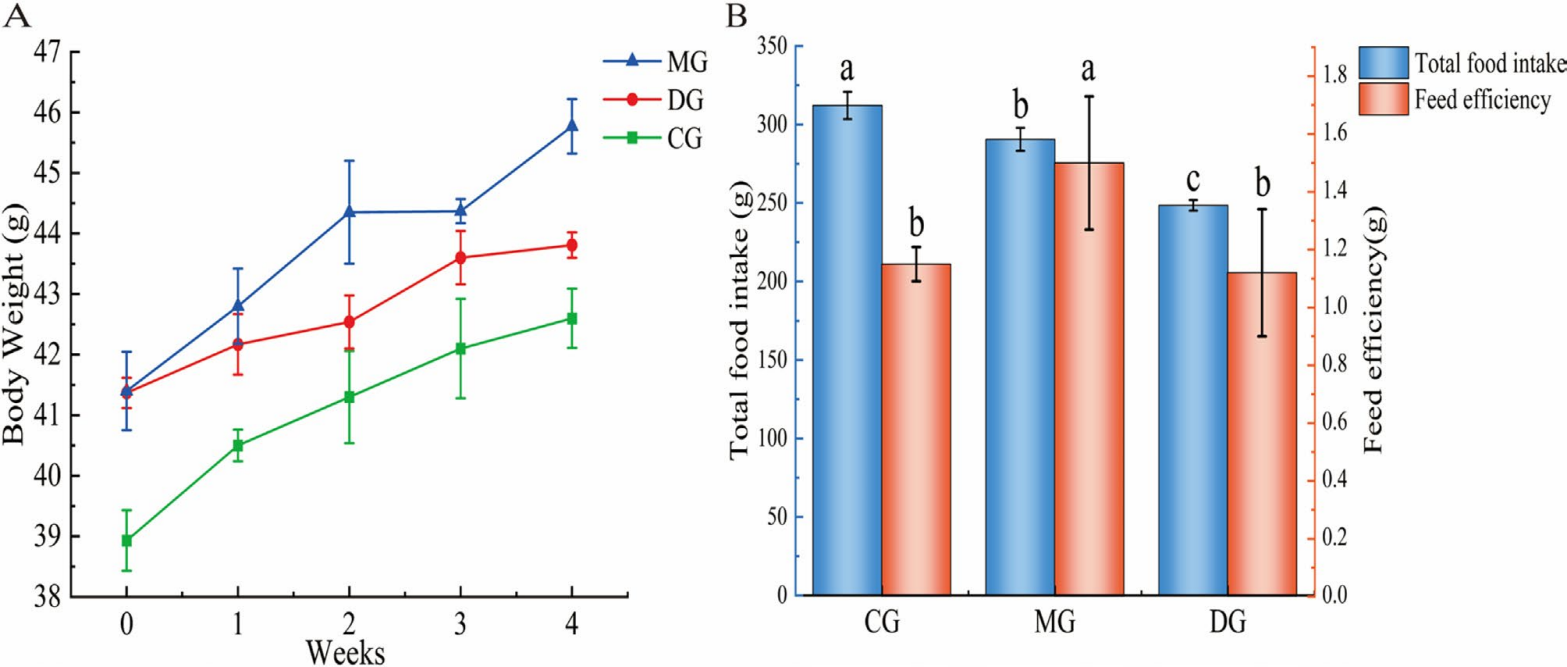
Effect of HAS on the body weight, food intake, and feed efficiency of mice (*n* = 10). Lowercase letters indicate significant differences among the mice in each group (*p* < 0.05). “CG” represents the blank group, “MG” represents the high‐fat model group, and “DG” represents the hydroxy‐α‐sanshool dose group.

The growth of the mice was normal, with no deaths or signs of diseases. The most apparent indicator of obesity is an increase in body weight. As illustrated in Figure 1A, after 8 weeks on a high‐fat diet, the weights of the mice in the MG and HAS‐DG groups significantly differed from those in the CG group, which was fed a standard diet for the same duration. Specifically, the weights of the mice in the MG and DG groups were significantly higher than those in the CG (*p* < 0.05). The weight of the mice in the MG continued to increase, whereas the weight of the mice in the DG tended to increase. After the experiment, the weight of the mice in the MG increased by 7.44% compared with that in the CG (*p* < 0.05). Compared with those in the MG, the body weights of the mice in the HAS‐DG decreased by 4.28% (*p* < 0.05). The food intake and feed efficiency of the mice in each group are shown in Figure 1B. Compared with those in the CG, the food intake of mice in the MG decreased significantly (*p* < 0.05) by 6.95%, and the food efficiency increased significantly (*p* < 0.05) by 30.42%. These observations are associated with high‐fat diet feeding. It is plausible that dietary fat contributes to body weight gain, whereas the high fat content may enhance satiety, thereby leading to decreased food intake. Given the experimental limitations, no paired control groups were designed in the present study; thus, we cannot exclude the possibility that HAS administration may reduce food intake motivation in mice, which could account for the lower total calorie intake in the HAS‐DG group compared with the MG group. Compared with those in the MG, the food intake and feed efficiency in the HAS group significantly decreased (*p* < 0.05) by 14.46% and 25.33%, respectively. These results suggest an association among high‐fat diet feeding, HAS intervention, and alterations in body weight, food intake, and feed efficiency in mice. Notably, the observed effects are consistent with the regulatory role of the gut‐brain axis in energy metabolism and food intake, although further investigations are warranted to verify the causal relationship between these findings and the gut‐brain axis‐mediated mechanism.

### 3.2 Effects of HAS on Organ Indices in High‐Fat Mice

The organ index refers to the ratio of organ tissue weight to mouse body mass and can be used to assess the growth status of mice. The effects of HAS on the organ indices of the mice in each group are presented in Table 2; compared with those in the CG, the spleen index, kidney index, brain index, liver index, and heart index of the MG significantly increased (*p* < 0.05) by 14.60%, 13.25%, 13.98%, and 12.96%, respectively. Compared with those in the MG, the kidney index, liver index, and heart index in the DG decreased significantly (*p* < 0.05) by 18.47%, 10.57%, and 16.39%, respectively, while the brain index and spleen index also decreased, but not significantly (*p* > 0.05). In summary, AS intervention effectively ameliorated the organ indices of mice fed a high‐fat diet, a characteristic consistent with the regulatory pattern mediated by the gut‐brain axis. It is speculated that HAS has the potential to alleviate the abnormal elevation of partial organ indices in mice induced by high‐fat diet feeding.

**TABLE 2:** Effect of HAS on the organ indices of mice (*n* = 10).

Group	Brain index (%)	Liver index (%)	Renal index (%)	Heart index (%)	Spleen index (%)
CG	0.83 ± 0.10^b^	4.65 ± 0.39^b^	1.37 ± 0.06^b^	0.54 ± 0.07^b^	0.29 ± 0.05^a^
MG	0.94 ± 0.09^a^	5.30 ± 0.47^a^	1.57 ± 0.12^a^	0.61 ± 0.08^a^	0.27 ± 0.06^a^
DG	0.91 ± 0.11^a^	4.74 ± 0.48^b^	1.28 ± 0.16^b^	0.51 ± 0.06^b^	0.25 ± 0.05^a^

*Note:* The experimental data are expressed as the mean ± standard error (SD). Lowercase letters indicate significant differences among the mice in each group (*p* < 0.05).

### 3.3 Effects of HAS on Intestinal Hormone Secretion in High‐Fat Diet‐Fed Mice

**FIGURE 2:**
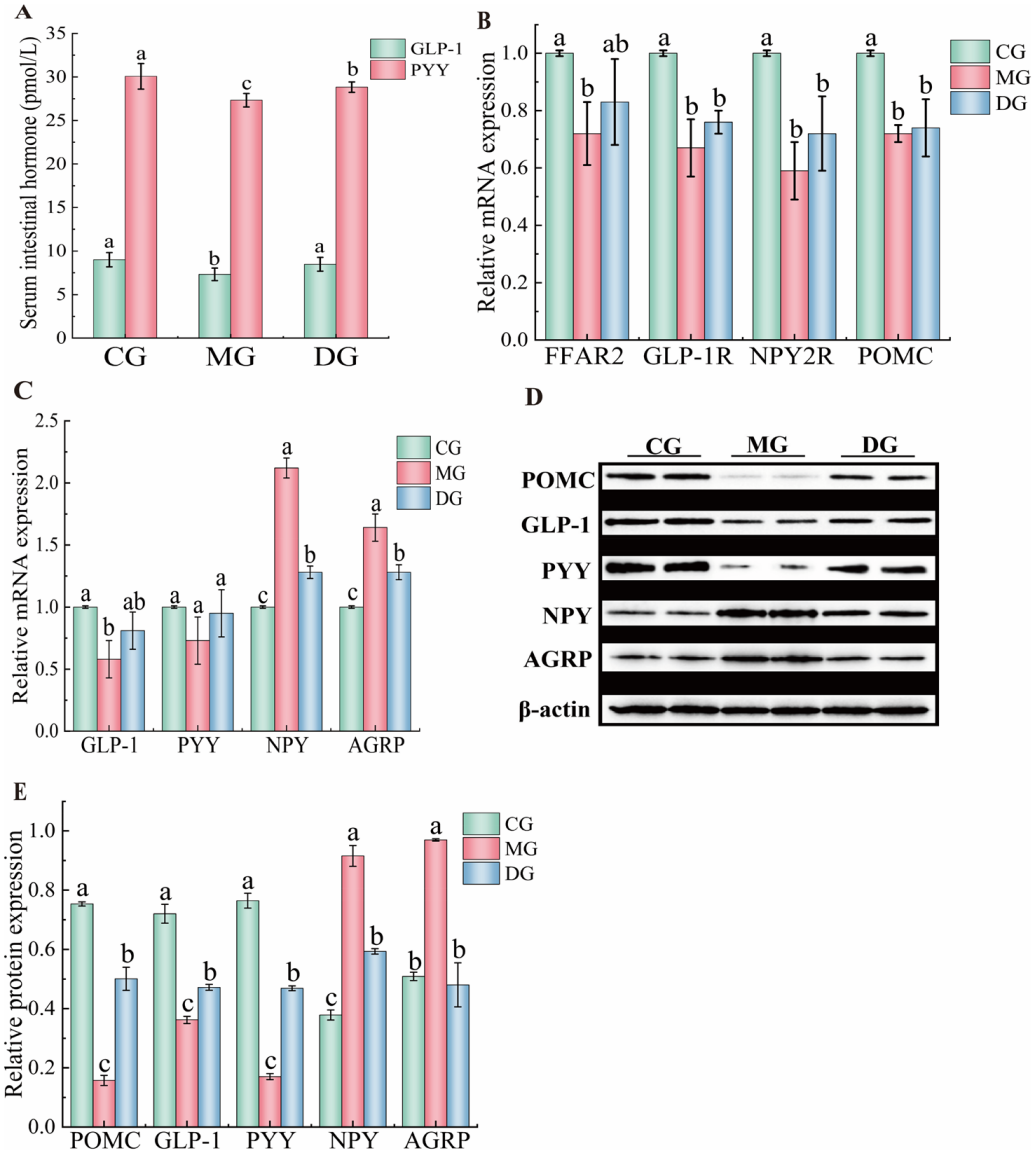
Effect of HAS on intestinal hormone secretion in high‐fat diet‐fed mice (*n* = 10). Lowercase letters indicate significant differences among the mice in each group (*p* < 0.05).

To investigate the effect of HAS on intestinal hormones in mice with high‐fat diet‐induced obesity, the serum levels of PYY and GLP‐1 and the mRNA expression of the FFAR2, GLP‐1, GLP‐1R, NPY2R, PYY, POMC, NPY, and AGRP genes in the small intestine were measured. The serum levels of the intestinal hormones PYY and GLP‐1 are shown in Figure 2A. Compared with those in the CG, the serum concentrations of the intestinal hormones GLP‐1 and PYY in the serum of mice from the MG decreased significantly (*p* < 0.05), by 18.56% and 15.69%, respectively. Compared with those in the MG, the GLP‐1 and PYY levels in the HAS group were significantly elevated (*p* < 0.05) after 4 weeks of intervention and increased by 9.11% and 5.49%, respectively, in the HAS group. The primary function of the small intestine is to absorb nutrients. The mRNA expression of appetite‐related genes in the small intestine is illustrated in Figure 2B,C. Compared with those in the CG, the relative mRNA expression levels of FFAR2, GLP‐1R, NPY2R, POMC, and GLP‐1 in the small intestine of mice in the MG decreased significantly (*p* < 0.05) by 28.01%, 33.00%, 41.11%, 28.05%, and 42.09%, respectively. The relative mRNA expression of PYY tended to decrease; however, this trend was not statistically significant (*p* > 0.05). In contrast, the relative mRNA expression of NPY and AGRP increased significantly (*p* < 0.05), with increases of 112.03% and 64.11%, respectively. Compared with the expression levels in the MG, the relative expression of FFAR2, GLP‐1R, GLP‐1, NPY2R, POMC, and PYY mRNA in the DG increased but not significantly (*p* > 0.05). The relative mRNA expression levels of NPY and AGRP decreased significantly (*p* < 0.05) by 39.62% and 21.95%, respectively. As illustrated in Figure 2D,E, the relative protein expression levels of POMC, GLP‐1, PYY, NPY, and AGRP in small intestine tissue were evaluated. Compared with those in the CG, the relative protein expression levels of POMC, GLP‐1, and PYY in the treatment group significantly decreased (*p* < 0.05) by 79.11%, 49.74%, and 77.84%, respectively, and the NPY and AGRP levels significantly increased (*p* < 0.05) by 141.99% and 90.53%, respectively. After the intervention with HAS, compared with those in the MG, the relative protein expression levels of POMC, GLP‐1, and PYY in the DG significantly increased (*p* < 0.05) by 217.66%, 30.36%, and 175.28%, respectively; however, the expression levels of NPY and AGRP significantly decreased (*p* < 0.05) by 35.22% and 50.47%, respectively. In summary, hydroxy‐α‐sanshool treatment significantly upregulated the mRNA and protein expression levels of GLP‐1 and PYY in the intestines of mice fed the high‐fat diet, markedly downregulated NPY expression, and simultaneously upregulated POMC expression. These results indicate that this compound can increase the secretion of intestine‐derived satiety peptides, which can then activate the hypothalamic satiety center either via vagal nerve conduction or direct entry into the systemic circulation (Yao et al. 2025). It is speculated that the enhanced intestinal satiety signaling induced by hydroxy‐α‐sanshool may be transmitted to the brain tissue through related signaling pathways, thereby regulating the expression of central neuropeptides and ultimately amplifying the satiety effect.

### 3.4 Effects of HAS on Brain Tissue in High‐Fat Diet‐Fed Mice

**FIGURE 3:**
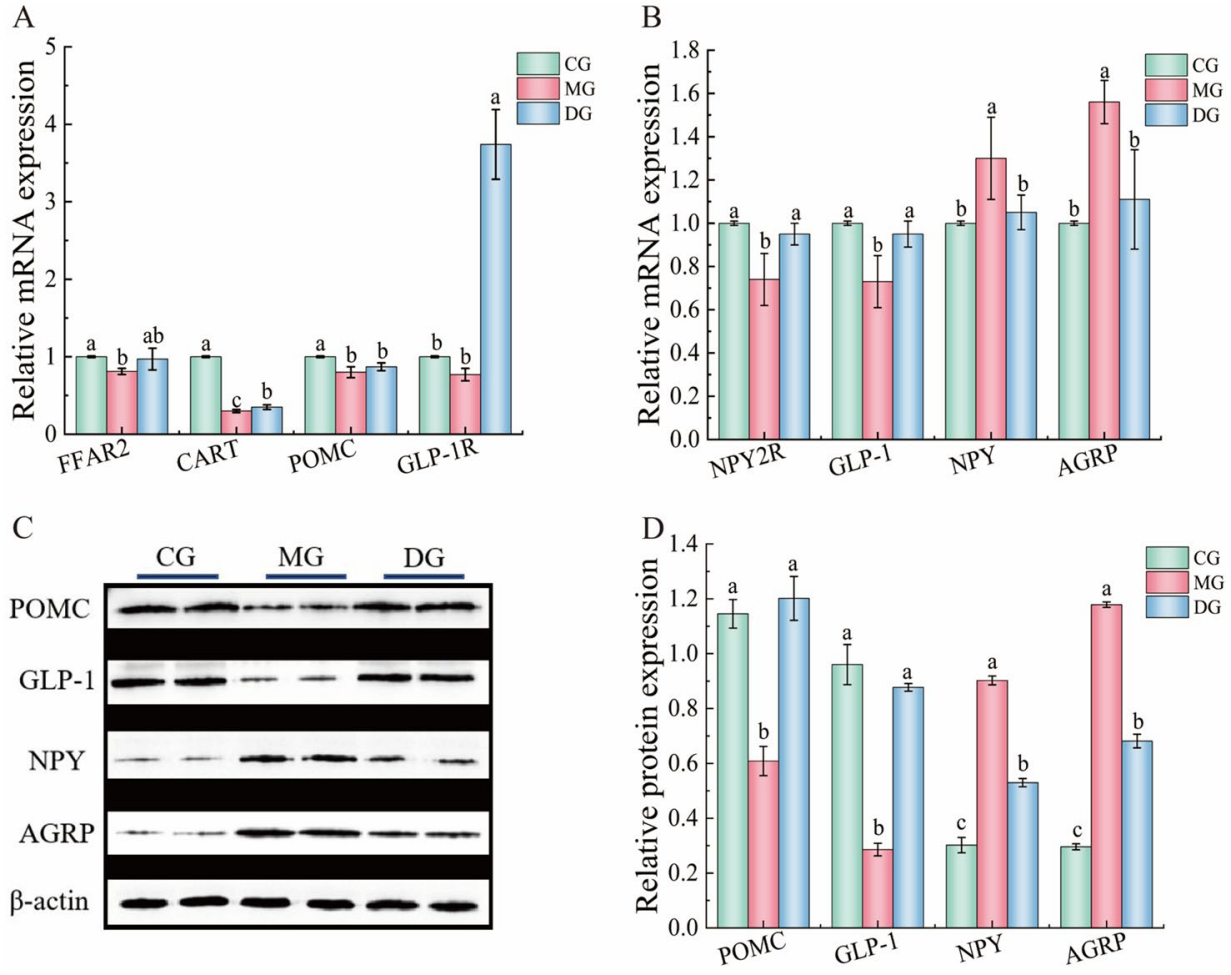
Effect of HAS on hypothalamic neurons in high‐fat diet‐fed mice (*n* = 10). Lowercase letters indicate significant differences among the mice in each group (*p* < 0.05).

The results of the detection of neurons responsible for regulating appetite in the brain tissue, such as CART, NPY, POMC, AGRP, and FFAR2, are presented in Figure 3A,B. Compared with those in the CG, the relative mRNA expression levels of FFAR2, CART, POMC, NPY2R, and GLP‐1 in the MG decreased significantly (*p* < 0.05) by 19.00%, 70.00%, 20.00%, 26.00%, and 27.00%, respectively. The relative expression of GLP‐1R mRNA decreased but not significantly (*p* > 0.05). The relative expression of NPY and AGRP mRNA significantly increased (*p* < 0.05) by 30.00% and 56.00%, respectively. Compared with that in the MG, the relative expression of CART, GLP‐1R, NPY2R, and GLP‐1 mRNA in the HAS group increased significantly (*p* < 0.05) by 16.67%, 385.71%, 28.38%, and 30.14%, respectively. The mRNA expression levels of FFAR2 and POMC increased with HAS intervention, but the difference was not statistically significant (*p* > 0.05). The relative expression of NPY and AGRP mRNA decreased significantly (*p* < 0.05) by 19.23% and 28.85%, respectively. These findings suggest that HAS may regulate the expression of appetite‐related gene mRNA in the brain tissue of mice with high‐fat diet‐induced obesity.

The effects of HAS on the relative protein expression of GLP‐1, NPY, AGRP, and POMC in the brain tissue of mice are illustrated in Figure 3C,D. Compared with the expression in the CG, the relative expression of POMC and GLP‐1 proteins in the MG decreased significantly (*p* < 0.05) by 46.87% and 70.25%, respectively, and the relative expression of NPY and AGRP proteins increased significantly (*p* < 0.05) by 198.80% and 292.91%, respectively. Compared with the expression levels in the MG, the relative protein expression levels of NPY and AGRP were significantly decreased by 42.28% and 42.19% (*p* < 0.05), respectively, under the HAS intervention. Moreover, the relative protein expression of POMC and GLP‐1 increased significantly, by 97.50% and 207.01%, respectively (*p* < 0.05). These results suggest that HAS may regulate the expression of appetite‐related genes in the brain tissue of mice with high‐fat diet‐induced obesity. In this study, central neuropeptides were measured in whole brain tissue without targeted analysis of the hypothalamus, a major limitation of this work. Future studies will focus on the hypothalamic arcuate nucleus to further elucidate the precise brain regions and cellular mechanisms underlying hydroxy‐α‐santalol‐mediated central satiety regulation.

### 3.5 Effects of HAS on the Intestinal Flora in High‐Fat Diet‐Fed Mice

#### 3.5.1 Alpha Diversity Analysis and Beta Diversity Analysis

**FIGURE 4:**
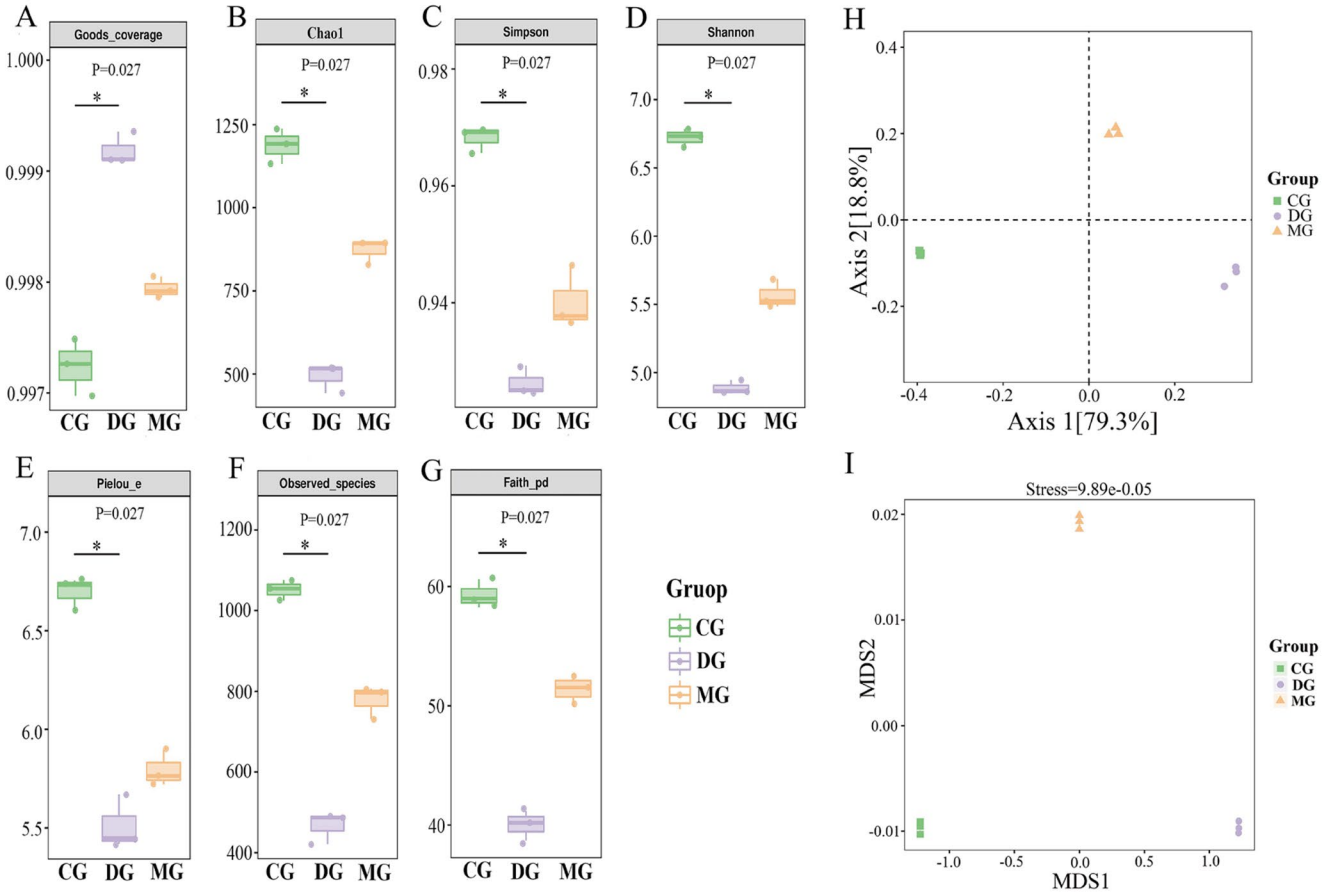
Effects of HAS on the *α* diversity of cecal contents in mice. Alpha diversity analysis revealed the Goods_coverage (A), Chao1 (B), Shannon (C), Simpson (D), Pielou_e (E), Observed_species (F), and Faith_pd (G) values of the intestinal flora of the mice in the CG, DG, and MG; analysis of beta diversity was performed using both PCoA (H) and NMDS (I). The figure is used to characterize richness with the Chao1 and observed species indices, diversity with the Shannon and Simpson indices, evolution‐based diversity with Faith's pd. Index, evenness with Pielou's evenness index, and coverage with Good's coverage index.

The alpha diversity index is a quantitative metric that can be used to assess differences in the richness and diversity of the gut flora between groups (Zhao et al. 2025). As demonstrated in Figure 4A, the Good coverage indices for all the groups exceed 0.99, indicating that these samples possess a reasonable number of sequences encompassing nearly the entire flora. As shown in Figure 4B–G, all indices in the DG mice were lower than those in the CG (*p* < 0.05), while all indices in the MG were greater than those in the CG. The results revealed a significant reduction in the abundance and diversity of intestinal flora in high‐fat diet‐fed mice, suggesting that long‐term dietary changes affect the overall structure of the intestinal flora (Geng et al. 2022). Following the HAS intervention, the species index, species evolutionary diversity index, and evenness index were elevated in the MG of mice compared with those in the DG. The results indicated that the high‐fat diet‐induced decrease in the diversity of intestinal flora in mice was effectively mitigated by HAS intervention, which increased the community richness and diversity of intestinal microorganisms in high‐fat diet‐fed mice. In the context of beta diversity analysis, both principal coordinate analysis (PCoA) based on linear microbial community structure and nonmetric analysis of microbial structure at multidimensional scales (NMDS) based on nonlinear microbial structure demonstrated that the samples from the three groups of mice were distinctly clustered within their respective groups, with a discernible trend of segregation between the groups (Figure 4H,I). These findings suggest that the composition of the intestinal flora was highly consistent among the groups of mice but differed from one group to another.

#### 3.5.2 Analysis of Species Composition and Function Prediction

**FIGURE 5:**
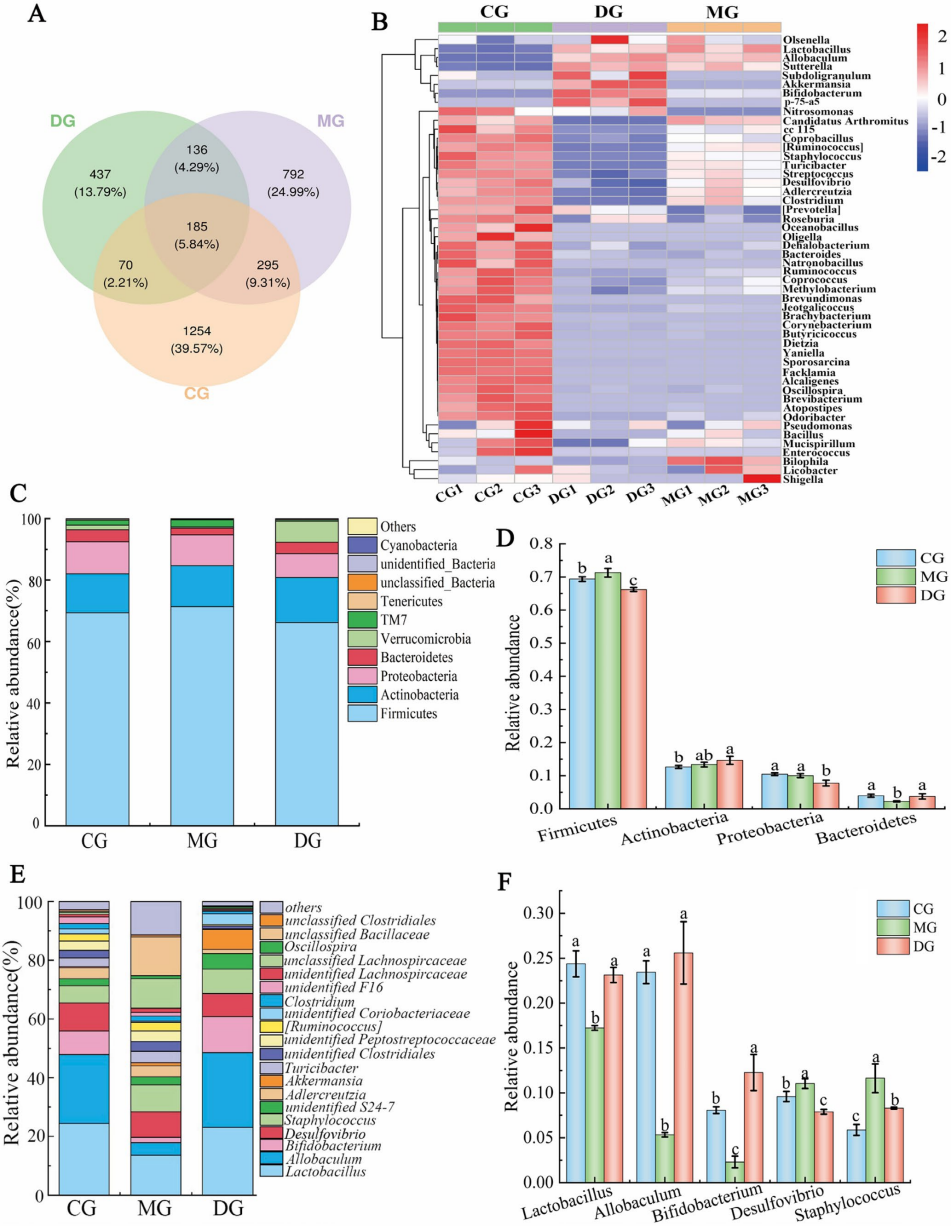
Species‐related abundance analysis at the genus level of the intestinal microbiota in mice (*n* = 10). Wayne plots of sample OTUs (A); a heatmap of species composition at the genus level for clustering of species (B); histograms of species‐associated abundance at the phylum level (C, D) and genus level (E, F). Lowercase letters indicate significant differences among the mice in each group (*p* < 0.05).

To further explore the alterations in the relative abundance of intestinal flora, Wayne plots of the CG, DG, and MG revealed a total of 185 OTUs (operational taxonomic units) in the three groups of mice, with 1254 OTUs unique to the CG mice, 437 OTUs unique to the DG mice, and 729 OTUs unique to the MG mice (Figure 5A). The species composition was then subjected to further analysis using heatmaps of the enriched data for the top 50 genera in terms of average abundance. Compared with DG mice, MG mice presented greater abundances of *Candidatus Arthromitus*, *CC_115*, *Coprobacillus*, *[Ruminococcus]*, *Staphylococcus*, *Turicibacter*, *Streptococcus*, *Desulfovibrio*, *Adlercreutzia*, *Clostridium*, and *Mucispirillum*, whereas the abundances of *Subdoligranulum*, *Akkermansia*, *Bifidobacterium*, and *p‐75‐a5* decreased (Figure 5B). The observed changes in abundance converged to the abundance of intestinal flora in the CG mice, suggesting that HAS intervention can increase intestinal flora richness in obese mice. An analysis of intestinal flora was conducted at the phylum and genus levels, with the objective of identifying species in groups of mice. The relative abundances of the phyla Firmicutes, Actinobacteria, Proteobacteria, and Bacteroidetes were the most abundant in the cecal content samples of the mice in each group (Figure 5C). The results of the relative abundance analysis of the four predominant phyla are presented in Figure 5D. The relative abundance of Firmicutes in the MG was significantly greater than that in the CG (*p* < 0.05), and the relative abundance of Firmicutes was significantly lower than that in the MG (*p* < 0.05) after HAS intervention. The relative abundance of Firmicutes in the MG was significantly lower than that in the MG (*p* < 0.05). Compared with that in the CG, the relative abundance of Bacteroidetes in the MG decreased significantly (*p* < 0.05), and the relative abundance of Bacteroidetes in the DG increased significantly (*p* < 0.05) after 4 weeks of intervention.

Among the top 20 genera, as illustrated in Figure 5E, the predominant genera of the mice in each group were *Lactobacillus*, *Allobaculum*, *Bifidobacterium*, *Desulfovibrio*, and *Staphylococcus*. As shown in Figure 5F, compared with those in the CG, the relative abundances of *Lactobacillus*, *Allobaculum*, and *Bifidobacterium* in the MG decreased significantly (*p* < 0.05) by 29.29%, 77.21%, and 71.50%, respectively. The relative abundances of *Desulfovibrio* and *Staphylococcus* increased by 15.00% and 97.89%, respectively (*p* < 0.05). Compared with the MG, the HAS intervention in the DG significantly increased the relative abundances of *Lactobacillus*, *Allobaculum*, and *Bifidobacterium* by 34.16%, 378.85%, and 433.48%, respectively (*p* < 0.05). Conversely, the relative abundances of *Desulfovibrio* and *Staphylococcus* decreased by 28.53% and 28.63%, respectively (*p* < 0.05). On the basis of the predictive annotation results from the KEGG database, metabolic pathways generally exhibited a higher average abundance and displayed relatively significant functional enrichment characteristics. As illustrated in Figure S2, the pathways involved included carbohydrate metabolism, amino acid metabolism, cofactor and vitamin metabolism, terpenoid and polyketide metabolism, and lipid metabolism. It should be noted that 16S rRNA gene sequencing only allows taxonomic identification at the genus level in most cases and fails to accurately distinguish differences at the species and strain levels. Therefore, future research will employ metagenomic sequencing combined with untargeted metabolomics to further resolve intestinal microbial strains and verify their actual functions at both the gene expression and metabolite levels, thereby remedying the deficiencies of 16S rRNA‐based functional prediction.

### 3.6 Effects of HAS on the Brain Tissue and Small Intestine Histopathology of High‐Fat Diet‐Fed Mice

**FIGURE 6:**
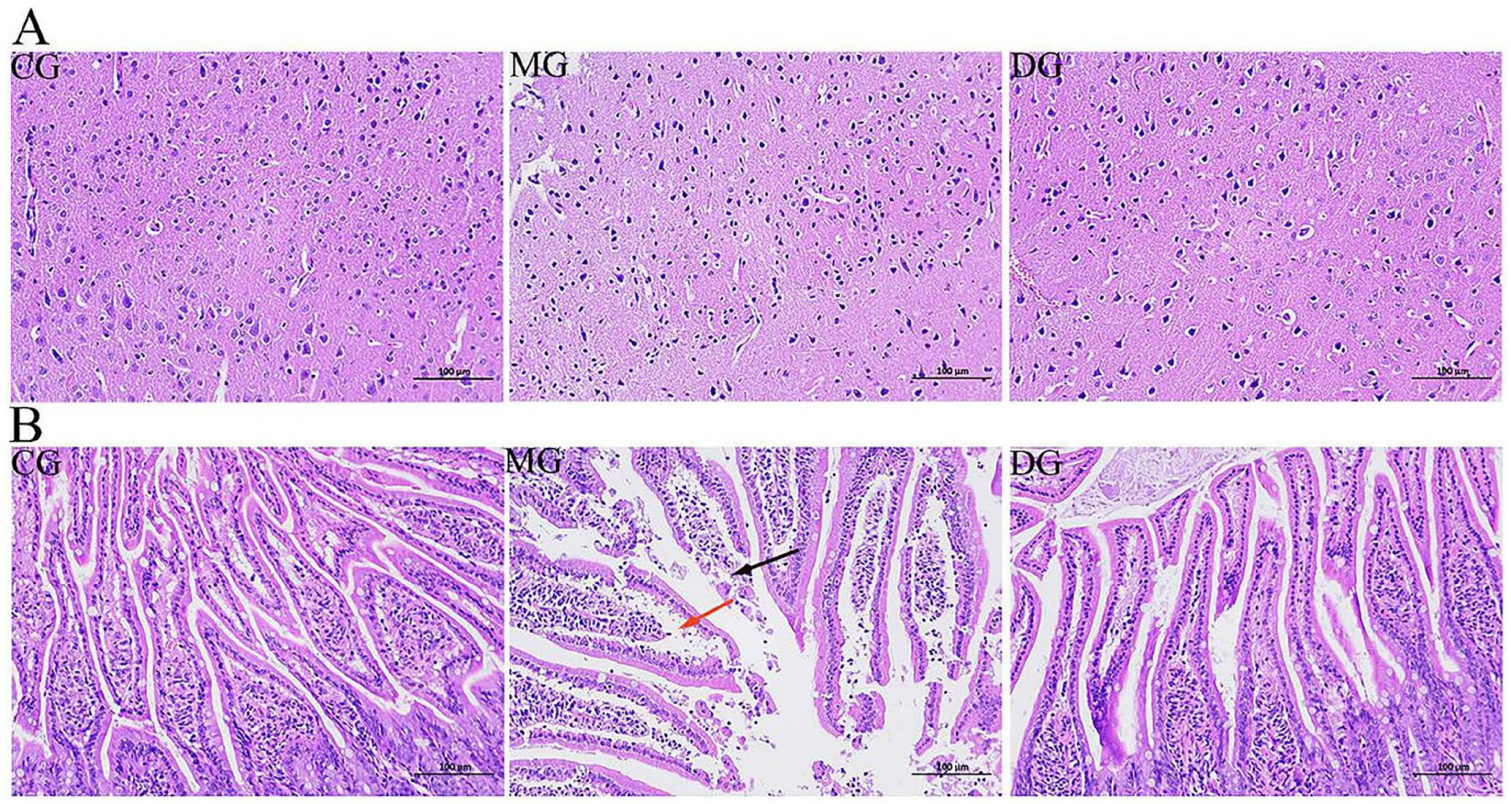
Effect of HAS on the morphology of brain tissue and small intestine tissue in mice (HE staining; scale bar: 100 μm). (A) HE staining of brain tissue; (B) HE staining of small intestine tissue.

In the CG, the number of nerve cells in the cortex of the brain tissue was abundant; these cells were clearly arranged, neatly defined, and clearly layered, and no obvious necrosis or inflammatory cells were observed. In the MG, the morphology of the brain tissue was poor, and a reduced number of constricted nerve cells were constricted, resulting in deep basophilic staining, which was an artifact caused by artificial squeezing, and no obvious necrosis or inflammatory cell infiltration was detected. The morphology of the brain tissue of the DG appeared to be similar to that of the MG but obviously improved (Figure 6A). In the small intestine of the CG, the intestinal villi were well developed, the mucosal epithelium remained intact, the glands were arranged neatly, the goblet cells were abundant, the submucosa and muscular layers were clear, and no obvious inflammatory cell infiltration was detected. In the MG, many mucosal epithelial cells were exfoliated, mucosal epithelial defects and a small number of goblet cells were lost (black arrow), the mucosal epithelium was separated from the lamina propria (red arrow), the lamina propria glands were arranged neatly, the submucosal structure was clear, the local muscular layer was missing, and no obvious inflammatory cell infiltration was detected. In the DG, intestinal villi developed, the mucosal epithelium was complete, the glands were organized neatly, the number of goblet cells was abundant, and the submucosa and muscular layer structures were clear, which greatly improved mucosal cell shedding and epithelial defects in obese people fed a high‐fat diet (Figure 6B).

## 4 Discussion

The development of obesity is quite complex and closely related to genetics, diet, exercise, energy metabolism disorders, and many other aspects (Jiang et al. 2024). In recent years, there has been a growing focus on the disparity between energy intake and consumption. Compared with those in nonobese individuals, multiple neuropeptides in the gastrointestinal tract and brain differ between obese individuals (Ghanemi et al. 2018). It has been demonstrated that the hypothalamus plays a pivotal role in the regulation of feeding, body mass, and energy metabolism (Buhmann et al. 2014). Notably, HAS may ameliorate obesity by modulating the gut microbiota, a finding that is potentially associated with the activation of the gut–brain axis signaling pathway.

The gut–brain axis is in constant communication and interaction through neural, endocrine, and immune signaling (Cryan et al. 2019). PYY and GLP‐1, two kinds of food‐inhibiting brain‐intestinal peptides, are hormones secreted by the intestinal tract. FFAR2 colocalized with L cells can stimulate L cells to secrete PYY and GLP‐1 in response to enteral nutrition, and its secretion is proportional to the body's caloric intake (Lafferty et al. 2018). GLP‐1 activates GLP‐1R through the intestinal paracrine system for energy balance and blood glucose control. Exogenous stimulation of GLP‐1R involves a series of physiological reactions, including decreased food intake, inhibition of gastric emptying, and increased insulin secretion stimulated by glucose (Hayes et al. 2010). PYY acts on the hypothalamus, induces satiety, and reduces food intake. Our results indicated that HAS treatment elevated FFAR2 mRNA levels in both the brain tissue and small intestine, which in turn stimulated L‐cells to secrete PYY and GLP‐1. Therefore, it is reasonable to speculate that the upregulation of FFAR2 expression may serve as a key molecular link underlying the HAS‐mediated promotion of gut‐brain peptide secretion.

AGRP/NPY neurons in the hypothalamic ARC are appetite‐promoting neurons, whereas POMC neurons are appetite‐inhibiting neurons (Seong et al. 2019). Food intake can be inhibited by the inhibition of NPY and POMC neurons that are activated later, and long‐term inhibition can be exerted through POMC signal transduction, which promotes satiety and reduces appetite (Bauer et al. 2015). Our results demonstrated that HAS treatment led to increased mRNA and protein expression levels of POMC in both the brain tissue and small intestine of mice, whereas the mRNA expression levels of NPY and AGRP were reduced. This positive and negative regulation can increase satiety and reduce appetite, which is consistent with the finding that the food intake of mice in the HAS DG is significantly lower than that of mice in the MG. These findings suggest that HAS might be capable of regulating the expression of appetite regulators in mice fed a high‐fat diet, which could in turn modulate food intake and maintain energy homeostasis. Nonetheless, the subject of discussion in our research bureau was limited to a single HAS content. In light of the findings of our present study, the effects of different doses of HAS on BGA in obese organisms should be investigated in future studies.

The intestinal microbial flora is the largest symbiotic system in the human body and environment and plays an extremely important role in human health and human diseases (Chakaroun et al. 2020). Many studies have shown that the abnormal composition of the intestinal microbial flora is related to the occurrence and development of human obesity and metabolic syndrome (Wang and Jia 2016). The activation of melanocortin 4 receptor (MC4R) in the intestinal endocrine system can stimulate the release of the satiety hormones GLP‐1 and PYY. Changes in the intestinal flora cause changes in serum GLP‐1 and PYY levels (Breton et al. 2016). Intestinal prebiotics can regulate the concentration of GLP‐1 and PYY secreted by intestinal endocrine cells, increase the satiety of the CNS to regulate food intake, and play a lipid‐lowering role in controlling obesity and related metabolic disorders (Neyrinck et al. 2012). Because the most important and basic function of the intestinal microflora is to obtain energy, eating habits strongly affect the structure and composition of the intestinal microflora. Studies have confirmed that the intestinal flora can affect host dietary behavior to a certain extent (Trevelline and Kohl 2022). Studies have shown that the intestinal flora of obese patients can promote the absorption of energy, resulting in the excessive accumulation of energy and weight gain (Liu et al. 2021). The abundance of *Firmicutes* in obese mice induced by high‐fat diet feeding increased significantly. Many studies have shown that compared with those of normal bodies, the composition and structure of the intestinal flora in obese bodies change significantly (Ley et al. 2006). Regulating the intestinal microbiome through diet, probiotics, antibiotics, surgery and fecal transplantation may significantly affect the obesity epidemic. Studies from animals and humans have proven the role of the intestinal microbiome in the occurrence and development of obesity (John and Mullin 2016). One study analyzed the intestinal flora composition of obese children and found that the proportion of *Firmicutes* increased, whereas the proportion of *Bacteroidetes* decreased (Indiani et al. 2018). In our study, the abundance of *Firmicutes* increased and that of *Bacteroidetes* decreased in the intestinal tract of mice fed HAS. *Bifidobacterium* and *Lactobacillus* are called probiotics and can regulate the acid–base balance in the intestinal tract and inhibit the growth of pathogens (Lee et al. 2020). In this study, compared with those in the MG, the relative abundances of *Bifidobacterium* and *Lactobacillus* in the cecal contents of mice in the HAS group increased. Studies have proven that the intestinal flora is related to the occurrence of insulin resistance and that an imbalance in the intestinal flora is closely related to abnormal glucose metabolism in the body. After the intestinal flora structure changes, the original gluconeogenesis process in the intestine and the degree of stimulation of the intestinal‐brain regulatory loop change, which reduces the sensitivity to insulin and results in insulin resistance.

In summary, the observed changes in hormones and gut microbiota in the present study are highly consistent with the hallmarks of energy homeostasis regulated by the gut‐brain axis and gut microbiota. Nevertheless, only statistically significant correlations were identified in this work, and definitive causal evidence remains to be established, which precludes definitive confirmation that HAS modulates feeding behavior and obesity‐related phenotypes in mice via the gut‐brain axis. In addition, individual confounding factors, including variations in food palatability and voluntary feeding motivation in mice, may contribute to fluctuations in food intake, which may potentially influence the precise evaluation of the independent regulatory effects of HAS on energy metabolism. Furthermore, only a single dosage of HAS was adopted in the current study, thus the dose–response relationship and optimal intervention concentration of HAS in regulating gut‐brain axis function and gut microbiota composition in obese mice remain to be elucidated. Finally, this study only detected differential abundance of gut microbiota at the phylum and genus levels, without systematic functional validation and integrated multi‐omics analyses. Accordingly, the specific role of gut microbiota in the HAS‐mediated gut‐brain axis signaling pathway has not been fully clarified, which limits the in‐depth mechanistic interpretation of the present findings. Collectively, our results demonstrate that HAS intervention effectively alleviates the abnormal phenotypes induced by high‐fat diet feeding in mice, including excessive body weight gain, reduced food intake, and elevated feed efficiency. These findings are in line with the regulatory properties of the gut‐brain axis in systemic energy metabolism and feeding control. Future investigations are warranted to adopt multi‐dose intervention regimens and perform integrated multi‐omics analyses focusing on the gut‐brain axis and gut microbiota, so as to further clarify the underlying causal regulatory mechanisms.

## 5 Conclusion

HAS can delay the weight increase caused by high‐fat diet consumption in mice, significantly reduce food intake and feed efficiency, and increase organ tissue enlargement. HAS modulates the expression of appetite‐related factors and reshapes the gut microbial community in high‐fat diet‐fed mice by regulating food intake, maintaining energy homeostasis, and ameliorating obesity‐associated indexes. These effects are highly consistent with the inherent mechanism by which the gut‐brain axis protects against obesity, indicating that the anti‐obesity action of HAS is closely associated with gut‐brain axis signaling. On the basis of the current findings, subsequent studies will focus on the dose–response relationship to identify the optimal effective concentration range and dose‐dependent effects of hydroxy‐α‐sanshool on the regulation of obesity‐related parameters. Furthermore, techniques such as gene knockout and fecal microbiota transplantation will be employed to verify the causal involvement of the gut‐brain axis and to identify key molecular targets that mediate the interaction between intestinal satiety signals and hypothalamic appetite regulation.

## Author Contributions

**Fangyan Xu:** conceptualization, methodology, data curation, formal analysis, writing – original draft. **Yuping Zhu:** data curation, supervision. **Tingyuan Ren:** funding acquisition, project administration, resources, writing – review and editing, supervision, visualization. **Jiao Xie:** software, investigation. **Likang Qin:** investigation, validation. **Huifang Chen:** software. **Tianting Luo:** writing – original draft, conceptualization, methodology, formal analysis, data curation.

## Funding

This research was supported by the China National Key R&D Program (Grant 2022YFD1100305). The Guizhou Science and Technology Department project (Guizhou Science and Technology Cooperation Foundation) (Grants [2022] Key 005 and ZK[2022]408).

## Ethics Statement

The study was conducted in accordance with the guidelines of the Declaration of Helsinki and approved by the Ethics Committee of Animal Experimentation at Gui‐zhou University (No. EAE‐GZU‐2020‐P003).

## Conflicts of Interest

The authors declare no conflicts of interest.

## Supporting information


**Figure S1:** HPLC chromatogram of HAS.
**Figure S2:** Plots of abundance of KEGG secondary functional pathways.

## Data Availability

Data will be made available on request.
