# Supplementary material for: Hydroxy‐α‐Sanshools From Zanthoxylum bungeanum Maxim. Alleviate Obesity in Mice via the Regulation of Appetite and Gut Microbiota
Source: Food Sci Nutr. 2026 Jun 1;14(6):e71952. doi: 10.1002/fsn3.71952 (PMC13239063; doi:10.1002/fsn3.71952)
Supplement: Supplementary file 1 — Figure S1: HPLC chromatogram of HAS. Figure S2: Plots of abundance of KEGG secondary functional pathways. [file FSN3-14-e71952-s001.docx]

**Supplementary material 1**

**FIGURE S**

*1 Purity detection of HAS*

The purity of HAS detected by high-performance liquid chromatography is shown in Fig. S1. The highest peak in the chromatogram is HAS, its retention time is 6.593 min, and the relative response value is more than 98%, indicating that the purity of HAS used in the experiment reaches the standard grade.


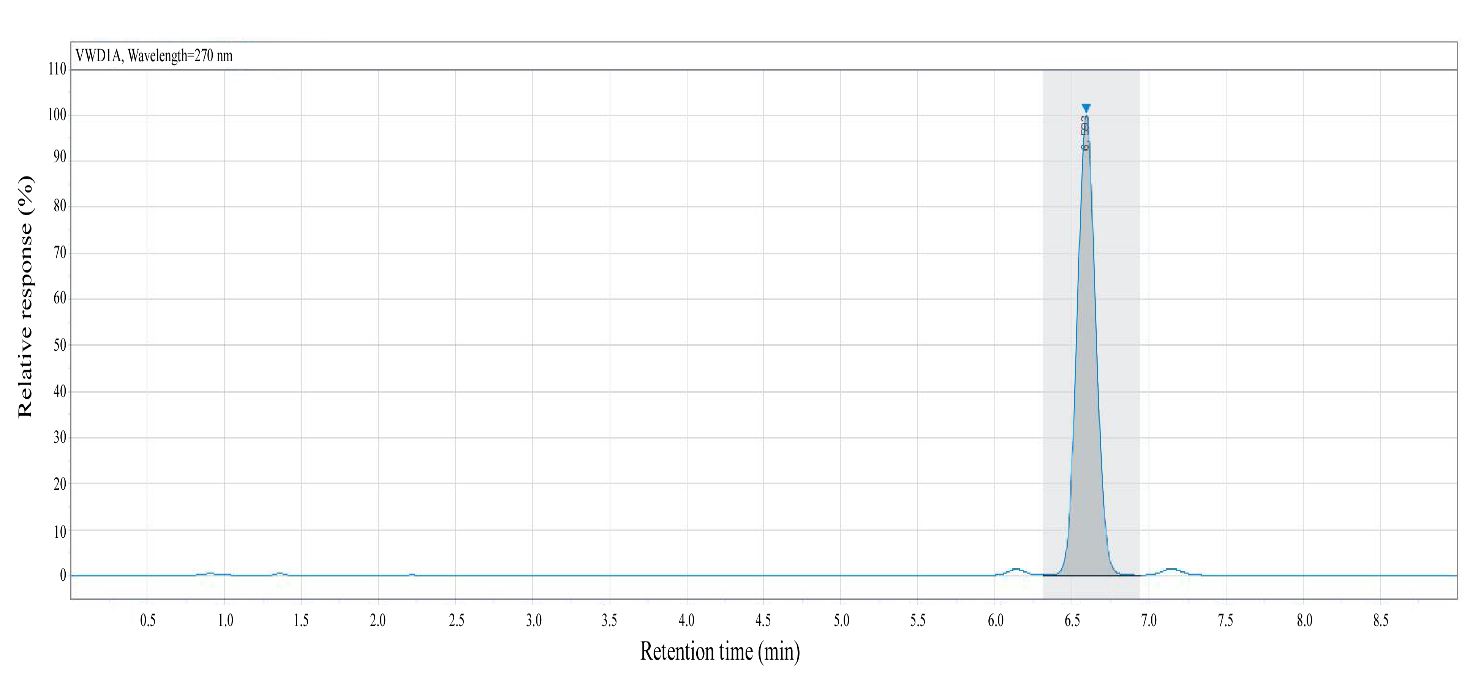
 **FIGURE S1** HPLC chromatogram of HAS


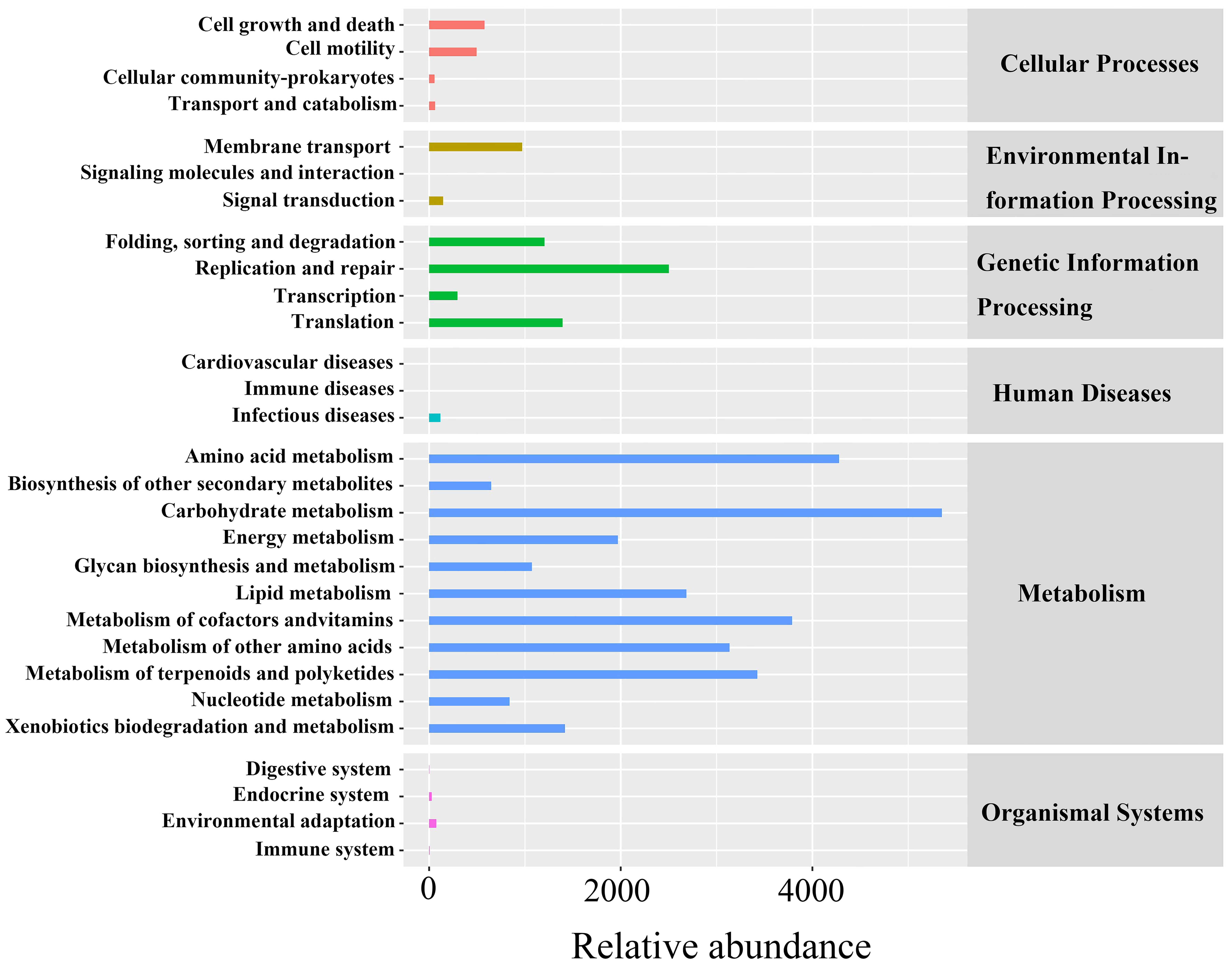


**FIGURE S2** Plots of abundance of KEGG secondary functional pathways

**Supplementary material 2**

**1、Experimental procedure**

**1.1 Total Microbiome DNA Extraction**

For microbiome samples from various sources, the optimal method for total DNA extraction was selected based on previous project experience. The DNA concentration was quantified using a Nanodrop spectrophotometer, and the quality of extracted DNA was assessed via 1.2% agarose gel electrophoresis.

**1.2Target fragment PCR amplification**

Target sequences that reflect the composition and diversity of microbial communities, such as microbial ribosomal RNA or specific gene fragments, were selected as amplification targets. Corresponding primers were designed based on the conserved regions of these sequences, and sample‑specific barcode sequences were appended accordingly. The variable regions (single or consecutive multiple regions) of rRNA genes or specific gene fragments were then amplified by PCR.

PCR amplification was performed using Pfu high-fidelity DNA polymerase (TransGen Biotech). The number of amplification cycles was strictly optimized to the minimum feasible value, while ensuring consistent amplification conditions across all samples in the same batch.

Negative controls were established throughout the PCR procedure to monitor microbial contamination derived from the environment and reagents. Any sample batch showing amplified bands in the negative controls was excluded from subsequent downstream experiments.

**1.3 Magnetic Bead Purification and Recovery of PCR Amplicons**

1.3.1 To 25 μL of PCR product, 0.8 volumes of magnetic beads (Vazyme VAHTSTM DNA Clean Beads) were added. The mixture was thoroughly suspended by vortexing and incubated on a magnetic stand for 5 min, after which the supernatant was carefully removed with a pipette. Subsequently, 20 μL of 0.8× magnetic bead washing solution was added. The solution was fully resuspended by vortexing, placed on a magnetic stand for 5 min of adsorption, and the supernatant was gently aspirated off.

1.3.3 200 μL of 80% ethanol was added, and the PCR tube was placed reversibly on the magnetic stand to allow the magnetic beads to adsorb onto the opposite side of the tube wall. After sufficient adsorption, the supernatant was carefully discarded.

1.3.4 The sample was incubated at room temperature for 5 min to allow complete ethanol volatilization until cracks appeared on the surface of the magnetic beads.

1.3.5 A total of 25 μL of Elution Buffer was added for DNA elution.

1.3.6 The PCR tube was placed on a magnetic stand for 5 min to achieve sufficient adsorption. The supernatant was then transferred to a sterile 1.5 mL centrifuge tube for preservation.

**1.4 Fluorescence Quantification of Amplified Products**

Fluorescence quantification of purified PCR amplicons was performed using the Quant-iT PicoGreen dsDNA Assay Kit, with a microplate reader (BioTek, FLx800) employed for detection. Based on the fluorescence quantification results, all samples were mixed in corresponding proportions according to the sequencing requirement of each sample.

**1.5 Sequencing Library Preparation**

Sequencing libraries were constructed using the TruSeq Nano DNA LT Library Prep Kit (Illumina, USA).

1.5.1 The terminal ends of the above amplified products were firstly repaired. The End Repair Mix 2 supplied in the kit was used to trim the overhanging bases at the 5' end of DNA fragments, meanwhile phosphorylating the 5' termini and filling in the missing bases at the 3' end.

1.5.2 An adenine (A) base was subsequently added to the 3' end of DNA fragments to prevent self-ligation and facilitate ligation with sequencing adapters, which contained a single overhanging thymine (T) base at the 3' terminus.

1.5.3 Sequencing adapters carrying library-specific index sequences were ligated to the 5' ends of DNA fragments, enabling the immobilization of DNA molecules on the flow cell.

1.5.4 BECKMAN AMPure XP Beads were used to remove adapter self-ligated fragments via magnetic bead screening and purify the adapter-ligated library system.

1.5.5 The adapter-ligated DNA fragments were subjected to PCR amplification to enrich the sequencing library templates. The enriched library products were further purified using BECKMAN AMPure XP Beads.

1.5.6 The library was finally subjected to fragment selection and purification via 2% agarose gel electrophoresis.

**1.6 High-throughput Sequencing on Platform**

1.6.1 Prior to sequencing, the constructed libraries were quality-checked using an Agilent Bioanalyzer with the Agilent High Sensitivity DNA Kit. Qualified libraries exhibited a single distinct peak without adapter contamination.

1.6.2 Library quantification was performed on the Promega QuantiFluor fluorescence quantification system using the Quant-iT PicoGreen dsDNA Assay Kit. Only libraries with a concentration above 2 nM were regarded as qualified.

1.6.3 Qualified sequencing libraries with non-repeating index sequences were serially diluted and mixed in appropriate proportions according to the required sequencing throughput. Subsequently, the mixture was denatured into single-stranded DNA using NaOH and loaded onto the platform for high-throughput sequencing.

1.6.4 Paired-end sequencing was performed on the MiSeq platform using the MiSeq Reagent Kit V3 (600 cycles). For paired-end sequencing on the NovaSeq platform, the NovaSeq 6000 SP Reagent Kit (500 cycles) was adopted instead.

Due to the relatively short read length of the MiSeq platform, the optimal sequencing length of target fragments was set at 200–450 bp to guarantee sequencing quality. Ribosomal RNA contains multiple conserved and highly variable regions. Primers are generally designed based on conserved regions to amplify single or multiple variable regions of rRNA genes, followed by sequencing and analysis of microbial diversity. Restricted by the read length of MiSeq sequencing and to ensure reliable sequencing quality, the optimal insert fragment size for sequencing was defined as 200–450 bp.

**2、Data Analysis Pipeline：**

①First, the raw off-machine data from high-throughput sequencing was preliminarily screened according to sequence quality, and problematic samples were re-sequenced and supplemented accordingly.

②Raw sequencing reads that passed the initial quality screening were assigned to corresponding libraries and samples based on index and barcode information, followed by removal of barcode sequences.

③Sequence denoising or OTU clustering was performed following the DADA2 pipeline in QIIME2 or the analytical workflow of the Vsearch software.

④The taxonomic composition of each sample (group) at different classification levels was visualized to reveal the overall community profile.

⑤The alpha diversity level of each sample was evaluated based on the distribution of OTUs across different samples, and rarefaction curves were plotted to assess the adequacy of sequencing depth.

⑥At the OTU level, the distance matrix of each sample was calculated. Multiple unsupervised ordination and clustering methods combined with corresponding statistical tests were adopted to evaluate the beta diversity differences and their significance among different samples or groups.

⑦At the taxonomic composition level, a variety of unsupervised and supervised ordination, clustering and modeling approaches combined with corresponding statistical tests were applied to further assess the differences in species abundance composition among samples or groups, and to screen for potential biomarker species.

⑧Based on the species composition and distribution across samples, correlation networks were constructed and topological indices were calculated to identify key microbial species.

⑨Based on the results of 16S rRNA gene sequencing, the metabolic functions of microbial communities in samples could be further predicted. Differential metabolic pathways were identified, and the species composition corresponding to specific pathways was also characterized.

1. **Bioinformatics Analysis**

**3.1 Raw paired-end sequencing data**

In this study, community DNA fragments were sequenced using the paired-end strategy on Illumina MiSeq and NovaSeq platforms. Raw sequencing data were stored in FASTQ format (paired Read 1 and Read 2 files, designated as R1.fastq and R2.fastq, respectively). The sequencing quality score for each base was recorded accordingly (see [https://en.wikipedia.org/wiki/Fastq](https://en.wikipedia.org/wiki/Fastq) for details).

**3.2 DADA2 Sequence Denoising**

Analysis Software: QIIME 2 (Version 2019.4) was used for bioinformatic analysis.

Analytical Procedures: First, the qiime cutadapt trim-paired command was used to trim primer sequences from paired-end reads, and reads without matched primers were discarded. Subsequently, the qiime dada2 denoise-paired workflow was implemented to perform quality control, sequence denoising, read merging, and chimera removal. The above procedures were conducted independently for each library. After denoising of all libraries was completed, amplicon sequence variant (ASV) representative sequences and the ASV table were merged, and singleton ASVs (ASVs with only one sequence count across all samples) were removed by default.

**3.3 Statistics of Sequence Length Distribution**

The length distribution of high-quality sequences obtained from all samples was statistically evaluated using custom R scripts.

**3.4 Taxonomic Annotation**

Bioinformatic analysis was performed using QIIME 2 (version 2019.4). For the 16S rRNA genes of bacteria and archaea, the Greengenes database (Release 13.8; http://greengenes.secondgenome.com/) (DeSantis et al., 2006) was used as the default reference, and the Silva database (Release 132; http://www.arb-silva.de) was also available as an alternative.

Taxonomic classification was conducted via the “classify-sklearn” algorithm embedded in QIIME 2 (https://github.com/QIIME2/q2-feature-classifier). Briefly, the representative sequences of ASVs or OTUs were annotated using a pre-trained Naive Bayes classifier with default parameters in QIIME 2. For the NCBI nt database, the “BROCC” algorithm ( https://github.com/kylebittinger/q2-brocc#the-brocc-algorithm) was adopted. Specifically, sequence alignment against the nt database (or curated subsets of the nt database) was first performed using blastn; taxonomic annotation was subsequently generated by running the “brocc.py” script with recommended parameter settings.

**3.5 Rarefaction of the OTU Table**

An OTU abundance table was generated in the preceding analytical procedures. Since subsequent analyses required all samples to be standardized to an identical sequencing depth, further normalization of the OTU table was performed via rarefaction. This approach randomly subsampled an equal number of sequences from each sample to achieve a unified sequencing depth, thereby estimating the observable OTUs and their relative abundances at the designated depth. This normalization process is also referred to as table rarefaction.

Software: QIIME 2 (Version 2019.4).

Procedures: The qiime feature-table rarefy function was used to perform rarefaction. The rarefaction depth was set to 95% of the minimum sequence count among all samples.分析软件：QIIME2 (2019.4)

1. **Alpha Diversity Indices**

To comprehensively evaluate the alpha diversity of microbial communities, Chao1 and Observed species indices were used to characterize community richness; Shannon and Simpson indices were applied to reflect community diversity. Faith’s PD index was adopted to represent phylogenetic diversity, Pielou’s evenness index was employed to assess community evenness, and Good’s coverage index was used to evaluate sequencing coverage. The calculation formulas for all alpha diversity indices are available at <http://scikit-bio.org/docs/latest/generated/skbio.diversity.alpha.> html#module-skbio.diversity.alpha.

Software: QIIME 2 (Version 2019.4).

Procedures: Based on the unrarefied ASV/OTU table, alpha diversity analysis was performed using the qiime diversity alpha-rarefaction command with the parameters --p-steps 10, --p-min-depth 10, and --p-iterations 10. The minimum rarefaction depth was set to 10, and the maximum rarefaction depth (--p-max-depth) was defined as 95% of the minimum sequencing depth across all samples. Ten even depth intervals were selected between the minimum and maximum depths, and rarefaction was repeated 10 times at each depth. The mean values of the diversity indices at the maximum rarefaction depth were used as the final alpha diversity metrics by default.

1. **Beta Diversity Analysis**

Software: QIIME 2 (version 2019.4).

Procedures: Using the rarefied OTU table, the qiime diversity core-metrics-phylogenetic command was executed to calculate four types of community dissimilarity distance matrices. Principal Coordinate Analysis (PCoA) was subsequently performed based on these distance matrices, and the results were exported as QZV format files. Visualization was achieved by uploading the generated QZV files to the QIIME 2 online viewer (https://view.qiime2.org/).

**6、**Sample coding was performed using a “random blinded method”. Both experimental operators and sequencing analysts were kept unaware of the grouping and treatment information of the samples. All samples were subjected to sequencing and subsequent bioinformatic analysis with anonymous codes throughout the entire process, which effectively avoided subjective bias and ensured the objectivity and reliability of the results.
